# Should Nano-Particles be Weighed or Counted? Technical Considerations to In Vitro Testing Originated from Corpuscular Nature of Nano-Particles

**DOI:** 10.1007/s00005-021-00623-8

**Published:** 2021-08-03

**Authors:** Wojciech Kałas

**Affiliations:** Hirszfeld Institute of Immunology and Experimental Therapy, Polish Academy of Sciences, Rudolfa Weigla 12, 53-114 Wrocław, Poland

**Keywords:** Nanoparticles, Dosing, Nano-compounds, In vitro, Cytotoxicity, Particulate matter, Inorganic nanoparticles

## Abstract

The abundance of nanoparticles introduced to household products created the great expectations towards the application of nanotechnology in biology and medicine. That calls for cost-effective preliminary assessment of its cytotoxicity and biological activity. There are many attempts for creating proper guidance and standards for performing studies regarding nanoparticles. But still some important aspects crucial for in vitro testing of nanomaterials need more attention. Particulate nature is an obvious and widely unappreciated property of nanoparticles. In the context of in vitro studies, this property is critical, and it should be, but rarely is, considered when designing, performing, describing or interpreting the experiments involving the solid nanoparticles. First, we should be aware of relatively small and limited number of nanoparticles in the experimental setup. Even crude estimation of its number will be useful for proper interpretation of results. Second, we should not presume even distribution of particles in the solution, moreover we should expect that sedimentation and aggregation play an important role in interactions of nanoparticles with cells. In that case, expressing the dose in mass/volume units may lead as astray. Finally, the relation of size, weight, and number of nanoparticles makes comparisons of activity of nanoparticles of different sizes very complex. Estimations of number of nanoparticles in the dose should be an integral part of experiment design, its validation and interpretation.

## Introduction

We are living at the dawn of the nanotechnology revolution. The number of new nanomaterials designed for professional and household applications grows rapidly every year (Hobson et al. 2016). Nanoparticles have relatively large area comparing to its overall mass. Thus, most of the atoms of small, few nanometer size nanoparticle are located on the surface (Naito et al. 2018). This is a main reason of the unique properties of nanomaterials comparing to its similar micro and macro counterparts.

The great variety of nanoparticles are embedded in household products. Its presence rises our interest about the toxicity of nanomaterials used in consumer products (Gupta and Xie 2018; Wolfram et al. 2015). The additional source of our concern is the end life of the household products when nanomaterials will be released into the environment. Finally, we are aware of the presence of airborne particulate matter that can have a detrimental effect on health (Riediker et al. 2019).

The definition of nanomaterial is straightforward and says that it is a material that has one of its dimensions below 100 nm (Naito et al. 2018). Often, this definition is stretched to materials with dimensions above 100 nm, but less than 1 µm or materials that exhibit properties attributable to nanoparticles. For example, and smallest environmental probes PM2.5 are often regarded as nanoparticles (Babadjouni et al. 2018; Gratton et al. 2008). Nevertheless, problems described in this commentary review similarly or even more apply to bigger particles. Wide application of nanotechnology created the great expectations towards the application of nanotechnology in biology and medicine. Despite the large number of studies there are several nanoscale drugs that were developed enough to enter clinical studies (Mukhopadhyay 2019). The most promising group are organic nanoparticles, mostly small liposomes or micelles used to increase stability or bio-availability of known drugs (Anselmo and Mitragotri 2019). Such new nanoscale formulations of the well-known drugs can improve the efficacy and selectivity of such medicines. Liposomal formulations of Doxorubicin (Caelyx) or Amphotericin B are leading examples (Cheng et al. 2011; Kopeckova et al. 2019). The other widely studied group of nanoparticles are the inorganic nanomaterials of different chemical composition, surface properties, size, and shapes suited for use in biological systems due to its unique magnetic, luminescent or antibacterial properties. In this regard the iron, lanthanide, gold or silver nanoparticles are most extensively studied (Damasco et al. 2020). Notably, apart from iron nanoparticles, none of the inorganic were approved by responsible regulatory bodies (Bobo et al. 2016; He et al. 2019; Ventola 2017).

At first, most of the nanomaterials, especially made of chemically ambient materials were regarded as non-toxic. Now, with more nanomaterials synthesized and studied we are aware of complex interactions of nanoparticles with living organisms (Lewinski et al. 2008; Saifi et al. 2018; Wysokińska et al. 2016, 2019). Abundance of newly designed nanomaterials calls for cost-effective preliminary assessment of its biological activity and cytotoxicity. In this regard the methods based on in vitro cultures of cells or small organisms are the best option. These are fast, not expensive, easy to scale and do not rise the ethical concerns. The methodology applied in such studies reflects those that is successfully used to test activity and cytotoxicity of soluble drugs or other molecules (De Matteis and Rinaldi 2018; Hillegass et al. 2010; Oliveira et al. 2019). The usual workshop consists of a variety of the cell culture-based techniques, including cytotoxicity studies, assessment of expression of specific proteins and RNA and activity of cell signaling pathway. But unique properties of nanomaterials make the proper selection of conditions for ex vivo testing of its activity or cytotoxicity especially challenging. There is a great literature describing methodology that can be used for in vitro testing of nanoparticles (Azhdarzadeh et al. 2015; De Matteis and Rinaldi 2018; Saifi et al. 2018; Savage et al. 2019). Despite use of well-known methods, there is growing need for further standardization of procedures and methodology in studies regarding nanoparticles. The reason is poor reproducibility of results and difficulties of its comparison (Faria et al. 2018). The guidances are published for researchers and publishers to increase quality of studies and published data. They are indicating possible disturbances of standard procedures that can be introduced by nanomaterials and underline the need for proper description and reporting nanomaterial properties (Faria et al. 2018). But still some aspects important for in vitro testing of nanomaterials are barely discussed and need more attention. This opinion/review will concentrate on the particulate nature of nanoparticles as a unique property of insoluble nanoparticles (or air-derived environmental samples) and its consequences for dosing, testing and exerting in vitro cytotoxic effect. The particulate nature is an obvious and widely unappreciated property of nanoparticles. Especially, in the context of in vitro studies, this property is critical. It should be, but rarely is, considered when designing, performing, describing and interpreting the experiments involving the solid nanoparticles. Here are the main consequences of particulate nature of nanoparticles.

## They are Only Billions

Why we use molar but not weight-based units in chemistry? Mainly, because that the particular molecules interacts with each other. Chemical reactions are stoichiometric and the number of acting chemical species is expressed as moles. Thus, we use molar concentration to describe interactions of chemical compounds with each other. Similarly, in a larger scale, one molecule of inhibitor interacts (usually) with one active site of the enzyme to diminish its activity. The particular number of ligands interacts with the particular number of receptors to trigger a signaling pathway. In end-user applications we alter to mass measure, but mostly for the practical reasons. The nanoparticle is the most basic unit that interact with cells, organelles, cellular structures, receptors, molecules and each other. On the other hand, the nanoscale or micro-scale are not alike a molecular scale. In case of small molecules or ions the number of acting entities extend our imagination (1 mol as 6.023 × 10^23^). But, in the case of nanoparticles, we usually deal with a large, but limited number of particles. That numbers are smaller than we tend to think about. What numbers it would be? As there is no reliable direct method of counting of nanoparticles, usually we could try to estimate its number by calculations basing on data regarding density and geometry of nanoparticles, light scattering or solution absorbance (Alexander and Goodisman 2014; Austin et al. 2020; Minelli et al. 2018; Park et al. 2004). The density of solid nanomaterials varies from 1 to 20 g/cm^3^ (Toy et al. 2011). In case of simple nanomaterials, the density of nanoparticles is closely related with density of original material. In case of complex and new nanomaterials, it can be determined, for example, by volumetric centrifugation method. The geometry of particular nanoparticles can be visualized by electron microscopy. This method along with dynamic light scattering may be used for determination of dimensions of nanoparticles (Eaton et al. 2017). These data can be used for calculations using formulas presented in Table 1 (NanoComposix 2021). To imagine the outstanding divergence between the molecular and nanosized world we could calculate the molar mass of the particular nanoparticles, understand as a mass of 1 mol of particles (Table 2). In molecular scale the molar mass of are in the range of grams to kilograms (proteins). In nanoscale the molar mass of particles range from kilograms to megatons. It reveals the other property of nanosized world. While molecules differ in weight about few thousand-fold, the weight of nanoparticles varies in million-fold range.

**Table 1:** Formulas used for the estimation of volume, mass, molar mass of particles and number of particles in 1 µg

${\text{volume}}_{{{\text{sphere}}\;{\text{particle}}}} = \frac{3}{4} \times \Pi \times \left( {\frac{{{\text{diameter}}}}{2}} \right)^{3}$	${\text{mass}}_{{{\text{particle}}}} = {\text{volume}}_{{{\text{particle}}}} \times {\text{density}}_{{{\text{particle}}}}$
${\text{molarmass}}_{{{\text{particle}}}} = {\text{mass}}_{{{\text{particle}}}} \times 6.02214 \times 10^{23}$	${\text{particlenumber}}_{{(1\;{\upmu\text{g)}}}} = \frac{{1\;{\upmu\text{g}}}}{{{\text{mass}}_{{{\text{particle}}}} }}$

**Table 2:** Number of cells cultured in the standard plasticware, as based on the HeLa cell line (Green BioResearch LLC 2016)

Type of plasticware	Area	Number of cells	
	(cm^2^)	Seeding	100% confluent
96-well plate	0.32	10 × 10^3^	40 × 10^3^
24-well plate	1.9	30 × 10^3^	120 × 10^3^
12-well plate	3.8	100 × 10^3^	500 × 10^3^
6-well plate	9.5	300 × 10^3^	1.2 × 10^6^
100 mm dish	57	2.2 × 10^6^	8.8 × 10^3^
150 mm dish	145	5 × 10^6^	20 × 10^6^

For example, spherical gold nanoparticles have 19.3 g/cm^3^ density (Toy et al. 2011). The volume of a single 60 nm particle is 14.38 × 10^−15^ cm^3^. Thus single particle weight 2.18 × 10^−15^ g and there are about 1.31 × 10^9^ (namely billions) of 60 nm particles in 1 µg. To compare, the 1 µg contains 33.44 × 10^15^ molecules of water, 3.34 × 10^15^ of aspirin, 2.35 × 10^15^ of palmitic acid or finally 103.79 × 10^12^ of insulin (Table 3).

**Table 3:** The examples of densities of selected nanomaterials along with its number in 1 µg and calculated molar mass in comparison to other molecules

	Diameter (nm)	Density (g/cm^3^)	Weight of single molecule (g)	Molar mass (g/mol)	Particles per µg	References
Water	–	–	29.91 × 10^–24^	18.01	33.44 × 10^15^	
Aspirin	–	–	299.16 × 10^–24^	180.16	3.34 × 10^15^	
Palmitic acid	–	–	425.81 × 10^–24^	256.43	2.35 × 10^15^	
Insulin	–	–	9.64 × 10^–21^	5.808 × 10^3^	103.69 × 10^12^	
PBS-loaded liposomes	65	1.0	143.79 × 10^–18^	86,59 × 10^6^	6.95 × 10^9^	Toy et al. (2011)
Iodide-loaded liposomes	65	2.4	345.10 × 10^–18^	207.83 × 10^6^	2.90 × 10^9^	Toy et al. (2011)
Iron oxide nanosphere	60	5.1	546.80 × 10^–18^	347.36 × 10^6^	1.73 × 10^9^	Toy et al. (2011)
PS nanosphere	120	1.05	950.02 × 10^–18^	572.36 × 10^6^	1.05 × 10^9^	Minelli et al. (2018)
Gold nanosphere	60	19.3	2.18 × 10^–15^	1.31 × 10^9^	458 × 10^6^	Toy et al. (2011)
Silica nanosphere	2	1.11	4.65 × 10^–21^	2.80 × 10^3^	215 × 10^12^	DeLoid et al. (2014)
Silica nanosphere	20	1.11	4.65 × 10^–18^	2.80 × 10^6^	215 × 10^9^	DeLoid et al. (2014)
Silica nanosphere	200	1.11	4.65 × 10^–15^	2.80 × 10^9^	215 × 10^6^	DeLoid et al. (2014)
Iron oxide nanosphere	2	5.1	21.36 × 10^–21^	12.9 × 10^3^	46.8 × 10^12^	Toy et al. (2011)
Iron oxide nanosphere	20	5.1	21.36 × 10^–18^	12.9 × 10^6^	46.8 × 10^9^	Toy et al. (2011)
Iron oxide nanosphere	200	5.1	21.36 × 10^–15^	12.9 × 10^9^	46.8 × 10^6^	Toy et al. (2011)

Next, let ask: How such numbers are relevant to in vitro experimental setups? In cell culture studies the effect of nanoparticles on cell viability or metabolism depends primary on the direct interaction with the cells (Sabella et al. 2014; Wysokińska et al. 2016). Thus, the ratio of the nanoparticles and cells will be a relevant factor that affects the likeness of internalization of nanoparticles into the cells. The number of the cells used in in vitro studies vary depending on procedure and cell type. There is usually few to several thousand of adherent cells in a single experimental well. The number of adherent cell in confluent culture varies from 50 thousand to 20–30 millions of HeLa cells (Table 4) (Green BioResearch LLC 2016). When studying the nanoparticles, especially of high density and large we can easily deal with the dose that consist several nanoparticles per cell. Some researchers try to design nanoparticles to be specific to particular molecules or cell structures. We should remember that there is a limited number of such targets in cells. Depending on the type of the target molecules its number may vary from few thousands to few millions (Biggin 2011). It is estimated that there are about 10 million ribosomes with in the eukaryotic cell or 88,000 of MHC receptors on the fibroblast cell (Boulanger et al. 2018). What effects could we expect if a dose of a few hundreds or even thousands of nanoparticles per cell is applied if ribosomes or MHC molecules are meant to be affected? We could flip the question. Could we exclude the effect of nanoparticles on particular structure if so small dose was used in the studies?

**Table 4:** Variations of culture volume/bottom area ratio in different plasticware and volumes that can be used

Type of plasticware	Area	Total volume		½ of total volume		Minimal volume		Standard volume	
	(cm^2^)	(ml)	V/A	(ml)	V/A	(ml)	V/A	(ml)	V/A
96-well plate	0.32	0.36	**1.13**	0.18	**0.56**	0.1	**0.31**	0.2	**0.63**
24-well plate	1.9	3.4	**1.79**	1.7	**0.89**	0.38	**0.2**	1	**0.53**
12-well plate	3.8	6.9	**1.82**	3.45	**0.91**	0.76	**0.2**	1.5	**0.39**
6-well plate	9.5	16.8	**1.77**	8.4	**0.88**	1.9	**0.2**	2	**0.21**

This parameter reflects variations of the number of nanoparticles on the bottom when mass/volume concentration is used to describe the dose. Area, total volumes, minimal volumes as indicated by producers of standard plasticware

Actually, some studies recognize importance of number, but we should be aware of misreporting. In some studies dose of nanoparticles is expressed in molar concentration, but it regards to amount of original material, not number of nanoparticles (Chithrani et al. 2006). We need to appreciate that number of nanoparticles should be considered in experimental design and result interpretation. The number of acting entities should be related to the number of target cells, organelles, receptors, or molecules. The nanoparticles differ greatly in size and density. Thus, even crude estimations may be good enough to avoid missinterpretations originated from comparing activity of several-fold or hundreds-fold different number of particles. Relation of particle number and target entities should be an important part of interpretation of result.

## Many Small Make a Great

**Fig. 1:**
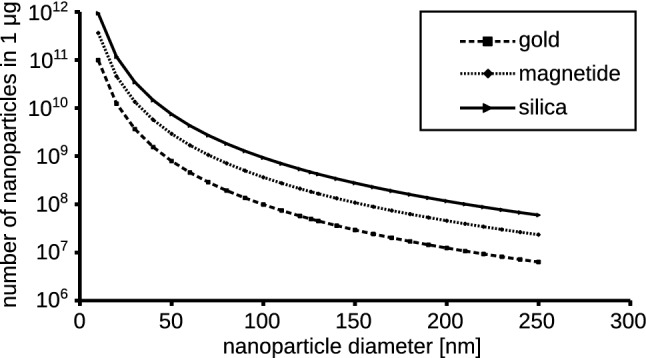
Size vs. particle number relation for spherical gold (19.3 g/cm^3^), magnetide (5.1 g/cm^3^) and silica (1.1 g/cm^3^). Particle diameter vs. calculated number in 1 µg mass is shown on the graph

One of the frequent questions asked in toxicology of nanoparticles is the question about impact of size or shape on the cytotoxicity (Gao et al. 2019; Toy et al. 2011; Wysokińska et al. 2016). The question that have very complex answer. Usually, the size/area ratio is indicated as a leading factor. It is correct, but for the whole understanding of nanoparticle activity the relation between the mass and nanoparticle count should also be considered. Attempts to verify the problem of size and shape in direct experiment led us and many researchers to experiment that two or more preparations of similar nanoparticles of different size are compared (Gratton et al. 2008; Wysokińska et al. 2016). The dose is usually defined in mass-related units and the number of particles rarely is regarded as a factor influencing the results. The size of a nanoparticle impacts its number in the mass unit in power of 3 (Table 1, Fig. 1). To illustrate this, if there is 10 × 10^9^ of the 20 nm nanoparticles in some mass, there would be only 10 × 10^6^ nanoparticles of 200 nm diameter in the same mass. In the case of spherical gold nanoparticles, there are only about 12 million 200 nm nanoparticles per µg. If we increase the size of the particle to 1 µm, the number of particles would decrease to about 100. Sometimes, our knowledge of nanoparticle interaction with biological systems is based on studies where the number of particles differed 140-fold, 300-fold or even 15,000-fold (Borm et al. 2018; Gratton et al. 2008; Mironava et al. 2010).

Similar reasoning should be applied to in vivo experiments. The same mass-related dose of different shapes of nanoparticles was administered. But differences in shape and size resulted in even tenfold difference in number of particles (Perry et al. 2017). How this fact impacts the penetration of tissues by studied compounds?

**Fig. 2:**
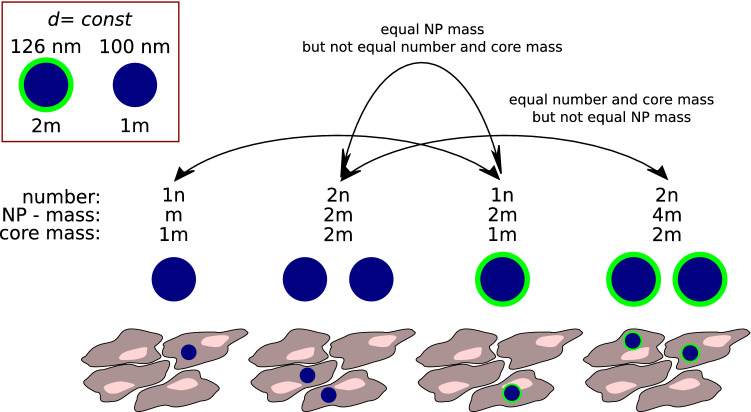
Graphical representation of problem of design and interpretation experiment with bare and and coated nanoparticles. If mass-related dose is used and coating increase size of particle then unequal number of particle is used and only portion of core material added to the experimental system. If 13 nm thick coating is applied on 100 nm nanoparticle then number of particles is reduced by half

The other problem often found in studies of nano-compounds is the question about impact of coating on the activity or cytotoxicity of the nanomaterial (Guo et al. 2016; Wysokińska et al. 2016). Usually, the toxic or harsh material is coated with ambient material like silica or polyethylene glycol polymer. Let think about spherical 100 nm material that was coated with 26 nm layer of silica derivative. And both the core and the coating have the same density. If we perform the experiment and compare weight related doses. Interpreting such an experiment is very challenging. If same weight related dose will be used, then we will compare cytotoxicity of a particular number of bare nanoparticles and half as much number of its coated derivative. Moreover, there is half as much of active/toxic component in mass unit. The second half is the coating (Table 5, Fig. 2). The concluding remark of such studies is that coating reduce toxicity of the nanoparticles (Ishida et al. 2020; Wysokińska et al. 2016). But can we be really convinced that the observed decrease in cytotoxicity is not because there is less toxic component and smaller number of particles in the sample? Should we expect half of dose will be similarly toxic as whole dose?

**Table 5:** Selected ratios of particle area and number of nanoparticles in arbitrary mass unit

Type of nanoparticle	Mass (m)	Diameter (nm)	Mass of the core (m)	Number in 1 m (*n*)	Area (a)
Spherical core nanoparticle	1	100	1	1	1
Spherical core nanoparticle	2	126	2	½	1.59
100 nm spherical core nanoparticle with 26 nm shell	2	126	½	½	1.59

Relation of mass and number in the bare and coated nanoparticles with assumption of equal densities of shell and core

## Truth Lies at the Bottom of a Well

In majority biological tests, especially in vitro, we test the soluble compounds. The standard procedure is that the tested compound is dispersed in the cell culture media that is later applied to the cell culture. Usually, the nanoparticles are tested in the same way (Azhdarzadeh et al. 2015; Patil et al. 2015; Savage et al. 2019). In that case, silent presumption of solubility or at least homogenic distribution leads us astray. Much guidance does not say anything about distribution of tested nanoparticles. Can we presume the even distribution of nanoparticles in the volume of the experimental well? No, we do not. All the inorganic nanoparticles are insoluble and have higher densities than the cell culture media and sediment on the bottom of the cells (Böhmert et al. 2018). What does it matter to us? It means that the place of interaction of adherent cells and the nanoparticles is defined rather by the area of the cell culture vessel than the volume of culture. Insolubility and sedimentation have further consequences. The dose should not be defined in mass/volume units as by changing the volume of culture, the dose is changed, too.

If in one procedure we apply 1 ml of 100 ng/ml of nanoparticles to 24-well plate and in the other procedure we add 2 ml of the same nanoparticles in the same 100 ng/ml concentration, the cells on the bottom will be interacting with twice as many nanoparticles (Table 4). Often, in the same project we study activity of nanoparticles using procedures that call for different scales and thus different plasticware. Switching from 96-well to 6-well dish and preserving mass/volume concentration could result in reduction of dose by three (Table 4). The overwhelming majority of studies regarding inorganic nanoparticles express dose in mass/volume units (Borm et al. 2018; Gnach et al. 2015; Murugadoss et al. 2017; Patil et al. 2015). Thus, without the detailed information about cell culture conditions like plastic type (area) and volumes the dose remains unknown and thus incomparable throughout the different studies. To make this matter even more complex. We really can not presume that all particle sediment on the bottom of the plate. In the case of 80 nm gold nanoparticles it can take as much as 120 h to sediment on the bottom (Alexander and Goodisman 2014). Moreover, some studies show that it takes about a week to deliver a full applied dose of 15 nm gold particles to the Caco-2 cells (Böhmert et al. 2018). But still, substantial part of the dose sediment in the first few hours (Böhmert et al. 2018). The sedimentation process can be modeled using effective density of NP, medium density and viscosity, and other parameters. But in that time cells should not be handled that is impractical in most procedures. Second, we can not presume that the mono-layer cells grow exclusively on the bottom of the well, some cells were shown to grow on the walls (Böhmert et al. 2018). The portion of such cell could be neglected in case of large culture plates or flasks, but in case of often used 96-well plate such cells can account for even 50% of cells (Böhmert et al. 2018).

The matter is even more complicated in case of non-adherent cell cultures. Cells and nano-compounds sediment with in different rate. In that case, defining the place of interaction and modeling of interaction of nanoparticles with suspension cell culture is even more difficult. In general, sedimentation is a huge problem. Most of the publications regarding nanoparticles use the mass/volume concentration as the measure of dose. The authors rarely include information about plastic-ware type and volumes used for the treatments. Without such information it is impossible to estimate the actual dose applied to the cells and thus compare the results and draw meaningful conclusions.

## Conclusions

The problem is clear, but the solutions are hazed. The first conclusion is that we should count nanoparticles. But we should ask a practical question. Can we count the nanoparticles? Even commercially available nanoparticles are not always counted and shipped with proper information. The existing calculators base on density and shape of nanoparticles for calculation of its number (NanopartZ 2021). But measurement of the density of newly developed material is also not standard procedure. The size of nanoparticles in the sample often vary, so we should be aware that our results are rather estimations than calculations (Schavkan et al. 2019). Nevertheless, the estimations should be made, and the aspect of actual number of particles studied should be present in experimental design and interpretation of results. In some experimental design, like coating studies the number of particles can be more important than mass or area of nanoparticles. Even crude estimation will be extremely useful for proper interpretation of results. The additional controls, consisting same number of particles should be included in studies. Second, we can not express the dose of insoluble nanoparticles in mass/volume units, especially in the studies performed on cells grown in mono-layer. By itself it says nothing. If we express the dose as mass/cm^2^ we will avoid variability of results that come from different cell culture conditions. If we want to compare the activity of different nanoparticles the number/area units seem to be the best option to express the dose. On the other hand, when the particle is soluble within the cell, the overall mass of the nanomaterial will be also important factor. Thus a good practice of reporting is vital (Faria et al. 2018). Moreover, we need to keep in mind that this is merely compromise that omit the time of sedimentation, cells growing on the walls or spontaneous aggregation (DeLoid et al. 2014). To overcome sedimentation process we could think about gentle spinning cells at beginning of procedure as it is done during some transfection protocols, but we need to be aware that such manipulation may additionally facilitate interaction of cells and nanoparticles.

Third, we as biologist need to be aware of the unique nature of material that our colleague chemist gave us for testing because our habits and standards may lead us astray. There is no such thing as a simple cytotoxicity test. The most important conclusion is that we should be always aware of the corpuscular nature of nanoparticles. The consequence that is its limited number of nanoparticles in the sample. Estimations of number of nanoparticles in the dose should be an integral part of experiment design and always should be included in interpretation of results, especially when comparing different sized and shaped nanoparticles.
